# A Solid-Contact Ion-Transfer Stripping Voltammetric Sensor for Reagent-Free Orthophosphate Determination in Tap Water

**DOI:** 10.3390/s26185722

**Published:** 2026-09-09

**Authors:** Anahita Izadyar, Scout Weatherford, Ezekiel McCain

**Affiliations:** Department of Chemistry and Physics, Arkansas State University, State University, P.O. Box 419, Jonesboro, AR 72467, USA

**Keywords:** orthophosphate, ion-transfer stripping voltammetry, electrochemical sensor, solid-contact sensor, PEDOT: PSS, tributyltin chloride, water quality monitoring

## Abstract

**Highlights:**

**What are the main findings?**

- A novel solid-contact ion-transfer stripping voltammetric (ITSV) sensor was developed for direct, reagent-free orthophosphate detection using a TBTC-based phosphate-selective membrane and a PEDOT: PSS solid-contact transducer.
- The sensor exhibited a linear response over 10–850 μM phosphate (R^2^ = 0.9955) with a limit of detection (LOD) of 2.8 μM and a limit of quantification (LOQ) of 9.4 μM, high selectivity toward phosphate, and successful application in tap-water samples.

**What are the implications of the main findings?**

- Ion-transfer electrochemistry provides a new strategy for the determination of non-redox-active phosphate without colorimetric reagents, enzymes, optical instrumentation, or extensive sample preparation.
- The developed platform enables rapid phosphate determination and demonstrates potential applicability for future real-time monitoring and offers strong potential for portable and autonomous water-quality monitoring applications.

**Abstract:**

Excess phosphorus loading is a major contributor to eutrophication and harmful algal blooms, creating a need for rapid, reliable, and field-deployable phosphate monitoring technologies. In this study, a novel reagent-free electrochemical sensor for orthophosphate determination was developed based on ion-transfer stripping voltammetry (ITSV) at a phosphate-selective membrane interface. The sensor consisted of a poly(vinyl chloride) (PVC)/o-nitrophenyl octyl ether (o-NPOE) membrane containing tributyltin chloride (TBTC) as a phosphate-selective ionophore integrated with a PEDOT:PSS solid-contact transducer for efficient ion-to-electron signal conversion. Orthophosphate was quantified through the controlled transfer of H_2_PO_4_^−^ ions across the membrane without the use of chemical reagents, enzymes, or redox mediators. Under optimized conditions, the sensor exhibited a linear response over the concentration range of 10–850 μM with high linearity (R^2^ = 0.9955) and a limit of detection of 2.8 μM. The corresponding limit of quantification (LOQ) was 9.4 μM. Chronoamperometric measurements provided rapid concentration-dependent responses over a broader range of 5–1000 μM, demonstrating the potential applicability of the platform for future real-time monitoring applications. The sensor also maintained strong analytical performance in tap water samples and in the presence of chloride, sulfate, and nitrate ions, with detection limits ranging from 3.8 to 4.7 μM. To the best of our knowledge, this work represents the first demonstration of a solid-contact ITSV sensor for direct orthophosphate determination and offers a simple, low-cost, and reagent-free approach for phosphate monitoring in aqueous systems.

## 1. Introduction

Phosphorus is an essential nutrient that regulates primary productivity in aquatic ecosystems. However, excessive phosphorus loading has become one of the primary drivers of eutrophication and harmful algal blooms (HABs) in freshwater and coastal environments [1,2,3]. Anthropogenic activities, including agricultural runoff, municipal wastewater discharge, industrial effluents, and urban stormwater inputs, introduce substantial amounts of phosphate into surface waters. Elevated phosphate concentrations promote the excessive growth of algae and cyanobacteria, resulting in oxygen depletion, biodiversity loss, degradation of ecosystem services, and deterioration of drinking-water quality [1,2,3]. Furthermore, many bloom-forming cyanobacteria produce potent toxins, such as microcystins and cylindrospermopsin, which pose significant risks to human and ecosystem health [4,5]. Consequently, the development of reliable and timely phosphate monitoring technologies has become increasingly important for eutrophication management, HAB prevention, and environmental protection.

Among the various phosphorus species present in natural waters, orthophosphate is considered the most bioavailable form and is often the limiting nutrient controlling algal productivity in freshwater ecosystems [6]. Because elevated phosphate concentrations frequently precede bloom development, monitoring orthophosphate can provide an early warning of nutrient enrichment before visible bloom formation or toxin production occurs [7]. Early detection enables proactive watershed management and nutrient-control strategies, reducing both environmental and economic impacts associated with HAB outbreaks. Consequently, monitoring systems capable of providing rapid, sensitive, and continuous measurements of orthophosphate are highly desirable for environmental surveillance applications.

Despite the importance of phosphate monitoring, most routine analyses remain dependent on laboratory-based methods. The molybdenum blue colorimetric method is widely regarded as the regulatory standard for phosphate determination because of its high sensitivity and analytical reliability [8,9]. In this assay, orthophosphate reacts with ammonium molybdate under acidic conditions to form a phosphomolybdate complex, which is subsequently reduced to generate a blue-colored species for spectrophotometric quantification [8]. Although highly accurate, this method requires chemical reagents, sample collection, laboratory instrumentation, and trained personnel. Moreover, delays associated with sample transportation and laboratory analysis limit their suitability for real-time environmental monitoring [10]. Automated nutrient analyzers partially address these limitations but remain relatively expensive, require significant maintenance, and consume substantial quantities of chemical reagents, hindering large-scale deployment in remote and autonomous monitoring systems [11,12].

Electrochemical sensing has emerged as a promising alternative owing to its portability, low power consumption, rapid response, and compatibility with miniaturized and wireless sensing platforms. However, direct electrochemical detection of phosphate remains challenging because orthophosphate is not intrinsically redox-active and therefore cannot be readily detected using conventional electron-transfer-based electrochemical techniques [13,14]. Most reported phosphate sensors rely on indirect detection strategies involving enzymatic reactions, metal-complex formation, catalytic redox processes, optical–electrochemical transduction, or potentiometric measurements [15,16,17,18]. Although these approaches have demonstrated useful analytical performance, many suffer from limitations including reagent dependence, biofouling susceptibility, limited operational stability, and decreased selectivity in complex environmental matrices.

Ion-transfer electrochemistry provides a fundamentally different strategy for the detection of non-redox-active ions. Rather than relying on electron-transfer reactions, ion-transfer techniques monitor the movement of ions across the interface between two immiscible electrolyte phases [13,14]. This approach has enabled sensitive electrochemical detection of numerous ionic species that are otherwise inaccessible using traditional voltammetric methods. Ion-transfer stripping voltammetry (ITSV) combines selective ion transfer with electrochemical preconcentration, resulting in enhanced sensitivity and low detection limits for ionic analytes [19,20,21]. Because the analytical signal originates from ion-transfer processes instead of faradaic redox reactions, ITSV offers an attractive route for the direct determination of orthophosphate without enzymatic amplification, derivatization chemistry, or reagent addition.

Recent advances in all-solid-state ion-selective electrode technology have further expanded opportunities for practical implementation of ion-transfer-based sensing systems. Conducting polymers, particularly poly(3,4-ethylenedioxythiophene): poly (styrene sulfonate) (PEDOT: PSS), have received considerable attention as ion-to-electron transducers because of their high capacitance, mixed ionic-electronic conductivity, excellent electrochemical stability, and compatibility with miniaturized electrode architectures [22,23,24]. The incorporation of a PEDOT: PSS intermediate layer between the sensing membrane and electrode substrate facilitates efficient charge transduction, minimizes potential drift, and suppresses interfacial water-layer formation, thereby improving long-term sensor stability and reproducibility [22,25]. These characteristics make PEDOT: PSS highly attractive for the development of robust solid-contact electrochemical sensors suitable for long-term environmental monitoring.

Graphite-based electrodes and other carbon-derived materials have also attracted considerable interest as low-cost alternatives for electrochemical sensor development because of their favorable electrical conductivity, ease of fabrication, and compatibility with miniaturized and disposable sensing platforms. Recent studies have demonstrated the utility of graphite-based materials in electrochemical sensing applications and their potential for portable environmental monitoring devices [26,27]. Although the present study employs glassy carbon electrodes to establish the proof-of-concept and fundamental analytical performance of the proposed sensing platform, future implementation on graphite-based and screen-printed electrode architectures may enable lower-cost and more scalable sensor deployment.

In this study, we report a novel reagent-free electrochemical sensing platform for orthophosphate determination based on ion-transfer stripping voltammetry at a phosphate-selective membrane interface. The sensing architecture consists of a poly (vinyl chloride) (PVC)/o-nitrophenyl octyl ether (o-NPOE) membrane containing tributyltin chloride (TBTC) as a phosphate-selective ionophore integrated with a PEDOT: PSS solid-contact transducer. The concentration-dependent response observed in this work is consistent with selective phosphate transfer into the membrane phase followed by voltammetric stripping to generate a quantitative analytical signal. Unlike conventional colorimetric or enzyme-based methods, the sensor operates without chemical reagents, enzymes, optical instrumentation, or extensive sample preparation.

To the best of our knowledge, this work represents the first implementation of ion-transfer stripping voltammetry for the direct electrochemical determination of orthophosphate using a solid-contact membrane electrode architecture. By combining selective phosphate ion transfer, electrochemical preconcentration, and PEDOT: PSS-mediated charge transduction, the proposed platform establishes a new strategy for reagent-free phosphate sensing and provides a foundation for future integration into low-cost screen-printed electrodes, wireless sensing platforms, and autonomous environmental monitoring networks for eutrophication management and HAB mitigation.

## 2. Experimental Section

### 2.1. Reagents and Materials

Poly(vinyl chloride) (PVC, high molecular weight), o-nitrophenyl octyl ether (o-NPOE), tributyltin chloride (TBTC), tridodecylamine (TDDA), tetrahydrofuran (THF), potassium dihydrogen phosphate (KH_2_PO_4_), potassium hydrogen phthalate (KHP), hydrochloric acid (HCl), potassium chloride (KCl), sodium chloride (NaCl), sodium nitrate (NaNO_3_), sodium sulfate (Na_2_SO_4_), and an aqueous dispersion of poly(3,4-ethylenedioxythiophene):poly(styrene sulfonate) (PEDOT:PSS) were purchased from Sigma-Aldrich (St. Louis, MO, USA) and used as received without further purification. All chemicals were of analytical-reagent grade or higher purity.

Ultrapure water (18.2 MΩ·cm) obtained from a Milli-Q purification system (Millipore, Burlington, MA, USA) was used for the preparation of all aqueous solutions. A 100 mM phosphate stock solution was prepared by dissolving KH_2_PO_4_ in ultrapure water and stored at 4 °C when not in use. Working phosphate standards were freshly prepared daily by serial dilution of the stock solution with the appropriate supporting electrolyte.

For ion-transfer stripping voltammetry (ITSV) measurements, phosphate standards were prepared in 0.1 M potassium hydrogen phthalate (KHP) supporting electrolyte. Chronoamperometric measurements were performed in KHP buffer adjusted to pH 6.0 using hydrochloric acid or potassium hydroxide as needed. Prior to analysis, tap-water samples were filtered through a 0.45 μm nylon membrane syringe filter (Millipore Sigma, Burlington, MA, USA) to remove suspended particulates.

To evaluate sensor selectivity, phosphate standards were prepared in the presence of common environmental interferents, including chloride, sulfate, and nitrate ions. These studies were conducted to assess the influence of background ions commonly found in natural waters on phosphate ion transfer and overall sensor performance.

### 2.2. Electrode Fabrication

#### 2.2.1. Fabrication of PEDOT: PSS Solid-Contact Electrodes

Glassy carbon electrodes (GCEs, 5 mm diameter) were used as the sensor substrates. Prior to modification, the electrode surfaces were mechanically polished using alumina slurries of decreasing particle size (1.0, 0.3, and 0.05 μm) on microcloth polishing pads. The polished electrodes were thoroughly rinsed with ultrapure water and sonicated sequentially in ethanol and ultrapure water for 5 min each to remove residual polishing particles and surface contaminants.

A PEDOT: PSS solid-contact layer was deposited onto the cleaned GCE surface by drop-casting 5–10 μL of an aqueous PEDOT: PSS dispersion. The coated electrodes were allowed to dry overnight under ambient laboratory conditions and subsequently placed under vacuum for 2 h to remove residual solvent and improve film adhesion and uniformity [28,29,30,31].

The PEDOT: PSS layer served as an ion-to-electron transducer between the conductive glassy carbon substrate and the ion-selective membrane. Owing to its high redox capacitance, mixed ionic-electronic conductivity, and excellent electrochemical stability, PEDOT: PSS enhanced charge-transfer efficiency, minimized potential drift, and improved signal reproducibility. Furthermore, PEDOT: PSS has been reported to reduce the formation of interfacial water layers and improve long-term stability in solid-contact ion-selective electrodes [22,23,24,25].

#### 2.2.2. Preparation of the Phosphate-Selective Membrane

The phosphate-selective membrane was prepared using a plasticized poly (vinyl chloride) (PVC) matrix containing tributyltin chloride (TBTC) as the phosphate-selective ionophore and tridodecylamine (TDDA) was incorporated as a lipophilic ionic additive to facilitate charge compensation within the membrane phase and improve membrane conductivity characteristics. Its primary role is to maintain favorable ion-transfer conditions within the membrane architecture. The membrane cocktail consisted of 64.4 mg PVC, 130 mg o-nitrophenyl octyl ether (o-NPOE), 3.0 mg TBTC, and 3.0 mg TDDA dissolved in 2 mL tetrahydrofuran (THF). The mixture was vortexed thoroughly and allowed to equilibrate for approximately 30 min to obtain a homogeneous solution.

A 6 μL aliquot of the membrane cocktail was drop-cast onto the PEDOT: PSS-modified electrode surface and allowed to dry overnight at room temperature, resulting in the formation of a uniform and mechanically stable membrane film. Prior to electrochemical measurements, the fabricated sensors were conditioned in a 1 mM phosphate solution (pH 4.0) for 12 h to establish equilibrium at the membrane-solution interface and improve analytical reproducibility.

The final sensor consisted of a glassy carbon electrode coated with a PEDOT: PSS solid-contact layer and an outer PVC/o-NPOE/TBTC/TDDA phosphate-selective membrane.

### 2.3. Electrochemical Instrumentation

All electrochemical measurements were performed using a CHI660D electrochemical workstation (CH Instruments, Austin, TX, USA) controlled by CHI software (version CHI660D). Experiments were carried out in a conventional three-electrode electrochemical cell consisting of a PEDOT: PSS/PVC-o-NPOE/TBTC/TDDA-modified glassy carbon electrode (5 mm diameter) as the working electrode, an Ag/AgCl (3 M KCl) electrode as the reference electrode, and a platinum wire as the counter electrode.

All measurements were conducted at ambient laboratory temperature (22 ± 2 °C). Before each experiment, test solutions were allowed to equilibrate to room temperature.

### 2.4. Ion-Transfer Stripping Voltammetry Procedure

Ion-transfer stripping voltammetry (ITSV) measurements were performed using a preconcentration step followed by a stripping voltammetric scan. Ion-transfer stripping voltammetry measurements were performed under fixed experimental conditions. The stripping scans were recorded over a potential range of 0.1 to 0.6 V versus Ag/AgCl at a scan rate of 50 mV s^−1^. Measurements were conducted in a cell volume of 5.00 mL under identical solution conditions. During the accumulation stage, dihydrogen phosphate ions (H_2_PO_4_^−^) were selectively transferred from the aqueous phase into the phosphate-selective membrane through complexation with tributyltin chloride (TBTC). Unless otherwise stated, a preconcentration time of 7 min was employed for all measurements.

Following the accumulation step, the potential was scanned to induce the back-transfer of phosphate ions from the membrane phase into the aqueous solution. The resulting stripping current was recorded and used as the analytical signal for phosphate quantification. Background voltammograms were obtained using phosphate-free supporting electrolyte and were subtracted from sample voltammograms to minimize background contributions and improve analytical accuracy.

Calibration measurements were carried out using phosphate standard solutions prepared in 0.1 M potassium hydrogen phthalate (KHP) supporting electrolyte. All voltammograms were recorded under identical experimental conditions, and the stripping peak current was plotted as a function of phosphate concentration to construct calibration curves. Calibration measurements were performed under fixed pH conditions using 0.1 M KHP supporting electrolyte, selected to provide stable phosphate-transfer responses during voltammetric measurements.

### 2.5. Chronoamperometric Measurements

Chronoamperometric measurements were performed to investigate the electrochemical response of the phosphate sensor under constant-potential conditions. Experiments were conducted in KHP buffer (pH 6.0) by stepping the potential to the desired value and recording the resulting current as a function of time.

Phosphate standard solutions of varying concentrations were analyzed under identical experimental conditions. The current-time responses were recorded following each potential step, and the steady-state current obtained after the decay of the capacitive charging current was used as the analytical signal for phosphate quantification.

All measurements were performed at room temperature using freshly prepared phosphate solutions. Each experiment was conducted in triplicate, and the reported data represent the mean values obtained from independent measurements.

### 2.6. Selectivity and Interference Studies

The selectivity of the phosphate sensor was evaluated in the presence of common inorganic anions frequently encountered in environmental and drinking water samples. Phosphate standard solutions ranging from 15 to 850 μM were prepared in matrices containing chloride (NaCl), sulfate (Na_2_SO_4_), or nitrate (NaNO_3_). Chloride, sulfate, and nitrate ions were introduced at fixed concentrations of 250 μM each, representing concentrations relevant to tap water matrices.

Ion-transfer stripping voltammetry (ITSV) measurements were performed under the same experimental conditions used for calibration studies. Background-corrected voltammograms were recorded for each solution, and the corresponding stripping peak currents were obtained. Calibration curves were subsequently constructed in the presence of each potential interfering ion and compared with those obtained in the supporting electrolyte and tap-water samples.

All measurements were conducted in triplicate using independently prepared solutions, and the reported values represent the mean ± standard deviation.

### 2.7. Tap-Water Sample Analysis

The applicability of the developed phosphate sensor was evaluated using tap-water samples to assess its performance in a real aqueous matrix. Prior to analysis, all samples were filtered through a 0.45 μm membrane filter to remove suspended particulate matter. No additional sample pretreatment, digestion, or derivatization procedures were performed.

Phosphate concentrations were determined using the standard-addition method to minimize potential matrix effects. Known amounts of phosphate standard solution were added directly to the filtered samples, and ion-transfer stripping voltammetry (ITSV) measurements were performed under the optimized experimental conditions described above. Phosphate concentrations were calculated from the corresponding standard-addition calibration plots.

## 3. Results and Discussion

### 3.1. Sensor Fabrication and Sensing Mechanism

The phosphate-selective sensor was fabricated by integrating a phosphate-selective PVC/o-NPOE membrane containing tributyltin chloride (TBTC) and tridodecylamine (TDDA) onto a PEDOT solid-contact layer deposited on a carbon electrode substrate. This multilayer architecture combines the high ion selectivity of the polymeric membrane with the efficient charge-transduction capability of the conducting polymer layer. PEDOT serves as an effective ion-to-electron transducer, facilitating charge transfer between the ion-selective membrane and the underlying electrode while minimizing interfacial resistance and potential drift.

**Scheme 1:**
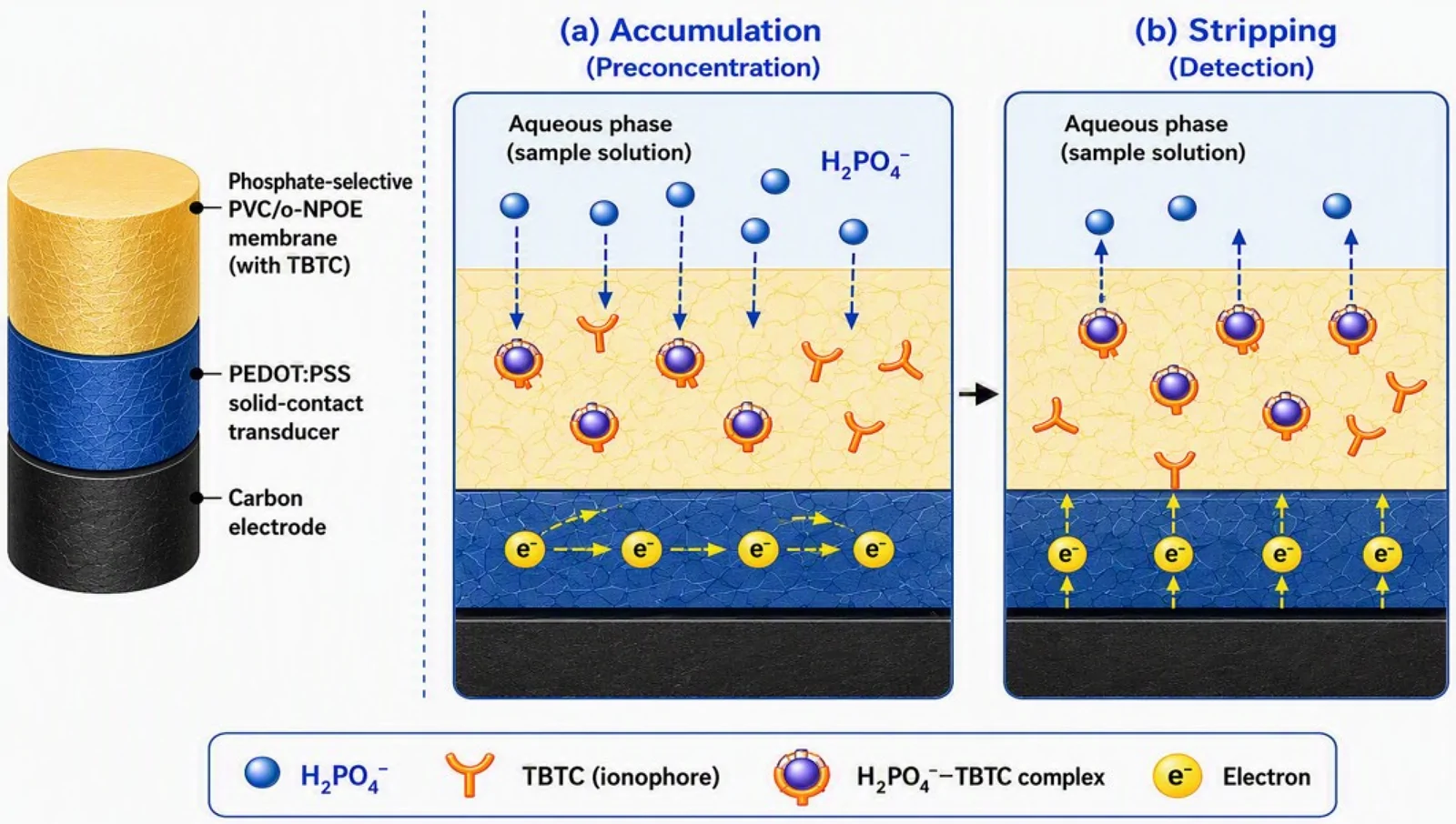
Schematic illustration of the phosphate ion-transfer stripping voltammetry (ITSV) sensor. The sensor consists of a glassy carbon electrode coated with a PEDOT: PSS solid-contact transducer and a phosphate-selective PVC/o-NPOE/TBTC/TDDA membrane. During the accumulation step, H_2_PO_4_^−^ ions are selectively extracted and preconcentrated within the membrane through interaction with the TBTC ionophore. During the stripping step, phosphate ions are released from the membrane, generating a current response proportional to phosphate concentration.

The proposed sensing mechanism is schematically illustrated in Scheme 1. During the accumulation step, dihydrogen phosphate ions (H_2_PO_4_^−^) in the aqueous phase selectively interact with the TBTC ionophore at the membrane/solution interface. The formation of the ionophore–analyte complex facilitates the transfer of H_2_PO_4_^−^ from the aqueous phase into the hydrophobic membrane, resulting in phosphate preconcentration within the sensing layer. Since ion transfer is governed by selective complexation with TBTC, competing ions exhibit significantly lower transfer efficiencies, thereby enhancing the overall selectivity of the sensor.

After the preconcentration step, the applied potential is scanned in the reverse direction, inducing the back-transfer (stripping) of phosphate ions from the membrane into the aqueous phase. This reverse ion-transfer process produces a measurable current that is proportional to the amount of phosphate accumulated within the membrane phase. Unlike conventional electrochemical detection methods that depend on redox reactions, the analytical response of this sensor is primarily attributed to selective ion-transfer processes occurring at the membrane interface. Therefore, the proposed platform enables direct electrochemical detection of orthophosphate, despite its intrinsic non-redox-active nature. The incorporation of the PEDOT intermediate layer plays a critical role in achieving stable sensor performance. The conducting polymer provides high capacitance and mixed ionic–electronic conductivity, enabling efficient charge compensation during ion-transfer processes. In addition, PEDOT helps minimize the formation of interfacial water layers, which are commonly associated with potential instability and signal drift in conventional coated-wire electrodes. The synergistic combination of selective phosphate ion transfer, electrochemical preconcentration, and efficient ion-to-electron transduction provides the basis of the proposed sensing strategy and enables sensitive, reagent-free phosphate detection. Because phosphate speciation depends on pH, the reported analytical performance corresponds specifically to the experimental conditions employed in this study. The relative abundance of phosphate species may influence sensor response under different pH conditions.

The proposed sensing mechanism is consistent with previous studies demonstrating the selective interaction of phosphate with tributyltin chloride (TBTC)-based membrane systems. Kim et al. [32] evaluated phosphate ion-selective membranes and cobalt-based electrodes for phosphate sensing applications. Glazier and Arnold [33] first demonstrated phosphate-selective polymer membrane electrodes, while Sasaki et al. [34] subsequently reported a highly selective phosphate ion sensor employing TBTC as the phosphate-recognition element. Therefore, the concentration-dependent stripping responses observed in the present work are consistent with established phosphate-selective membrane behavior reported in the literature.

### 3.2. Optimization of Experimental Parameters

The analytical performance of the sensor is influenced by operational conditions and membrane composition. The experimental conditions reported throughout this study correspond to the selected operating conditions used for analytical evaluation. While several parameters were considered during sensor development, the objective of the present work was to evaluate the performance of the optimized sensing platform rather than to provide a comprehensive optimization study.

Among the investigated parameters, accumulation time exhibited a significant effect on sensor sensitivity. Increasing the accumulation period resulted in higher stripping currents due to the greater amount of phosphate ions transferred into and retained within the sensing membrane. However, after reaching an optimal accumulation time, only minimal increases in signal intensity were observed, suggesting that the membrane approached its maximum phosphate uptake capacity. Therefore, excessively prolonged accumulation periods provide limited analytical improvement while increasing the overall analysis time.

The composition of the ion-selective membrane was also found to be a key factor affecting the voltammetric response. The ratio of PVC polymer matrix to o-NPOE plasticizer influences membrane flexibility, ion mobility, and analyte partitioning behavior. In addition, the concentration of TBTC directly determines the number of available phosphate recognition sites within the membrane. Low ionophore concentrations resulted in decreased sensitivity due to limited phosphate complexation, whereas excessive TBTC loading did not produce proportional signal enhancement and could contribute to increased background currents.

The incorporation of TDDA further enhanced sensor performance by improving ionic conductivity within the membrane and facilitating charge compensation during phosphate ion-transfer processes. Membrane thickness also significantly affected the stripping response. Thin membranes provided faster ion-transfer kinetics but limited phosphate accumulation capacity, whereas excessively thick membranes hindered ion diffusion and resulted in slower response times and broader stripping peaks. Thus, an optimized membrane thickness was selected to achieve a balance between high sensitivity and rapid analytical response.

The thickness of the PEDOT transducer layer also played an important role in sensor stability and reproducibility. An appropriately optimized PEDOT film reduced baseline drift and improved signal consistency through its high interfacial capacitance and efficient mixed ionic–electronic conductivity [22,23,24,25]. Overall, the optimization studies demonstrate that both the ion-selective membrane properties and the solid-contact architecture are essential for achieving enhanced analytical sensitivity, improved signal stability, and reliable phosphate detection.

The experimental conditions reported throughout this study correspond to the optimized operating conditions selected based on signal magnitude, peak shape, and reproducibility. The objective of the present work was to evaluate the performance of the optimized sensor rather than to provide a comprehensive parameter-screening study.

### 3.3. Voltammetric Response and Calibration Characteristics

**Figure 1:**
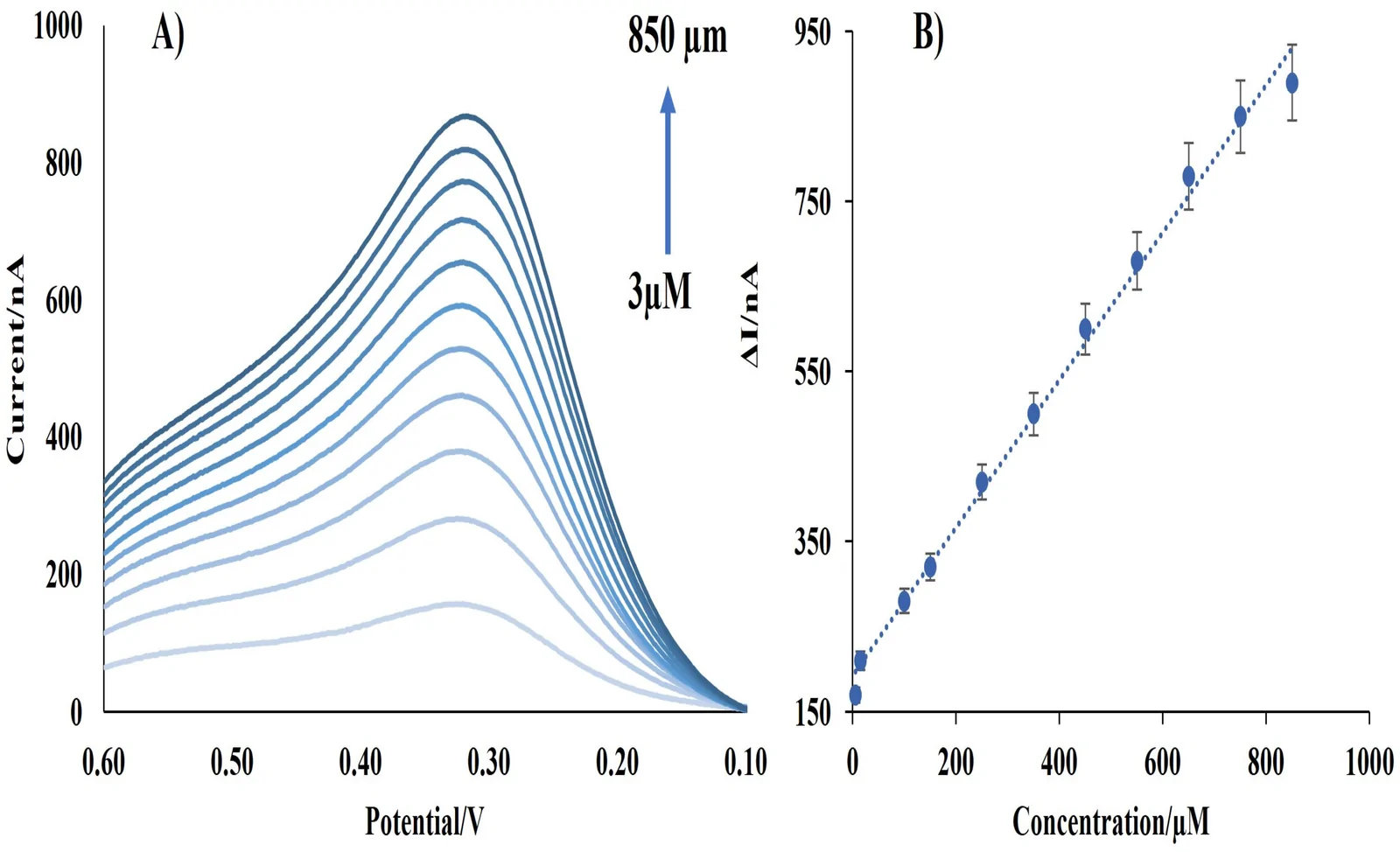
(**A**) Representative ion-transfer stripping voltammograms obtained for orthophosphate concentrations ranging from 10 to 850 μM using the PEDOT:PSS/PVC-o-NPOE/TBTC/TDDA sensor. (**B**) Background-subtracted LSV response expressed as ΔI (peak current minus background) and the corresponding calibration curve of stripping peak current versus orthophosphate concentration. Each data point represents the mean value of four replicate measurements (n = 4), and the error bars represent ± one standard deviation (SD). The sensor exhibited high linearity (R^2^ = 0.9955) and a limit of detection (LOD) of 2.8 μM. Measurements were recorded over a potential window of 0.1–0.6 V versus Ag/AgCl.

Representative background-subtracted ion-transfer stripping voltammograms obtained for phosphate concentrations ranging from 10 to 850 μM are shown in Figure 1A. Measurements were performed using a 7 min preconcentration step to facilitate phosphate accumulation within the TBTC-based ion-selective membrane prior to the stripping scan. Background voltammograms recorded in phosphate-free supporting electrolyte were subtracted from the analytical signal to minimize capacitive and nonfaradaic contributions.

As shown in Figure 1A, a well-defined stripping peak was observed at approximately 0.302 V, with the peak current increasing progressively as phosphate concentration increased. The peak potential remained essentially constant over the investigated concentration range, indicating a stable and reproducible ion-transfer process at the membrane-solution interface. The increase in stripping current reflects enhanced accumulation of H_2_PO_4_^−^ ions within the membrane during the preconcentration step, resulting in a greater amount of phosphate available for stripping during the voltammetric scan.

The corresponding calibration curve (Figure 1B), constructed from the background-corrected stripping peak currents, exhibited high linearity over the concentration range of 10 to 850 μM. Linear regression analysis yielded a correlation coefficient of R^2^ = 0.9955, demonstrating a strong relationship between phosphate concentration and analytical response. The calibration curves were generated from experimentally measured responses and were not constrained to pass through the origin. Small intercept values may arise from background correction procedures, residual capacitive currents, and instrumental noise.

The limit of detection (LOD), calculated using the 3σ/m criterion, was determined to be 2.8 μM, where σ represents the standard deviation of the blank response and *m* is the slope of the calibration curve. This detection limit is suitable for monitoring phosphate concentrations commonly encountered in natural waters, agricultural runoff, and wastewater effluents. The limit of quantification (LOQ), calculated as 10σ/m, was determined to be 9.4 μM. The reported calibration range was selected to provide reliable quantitative measurements across the investigated concentration interval.

All calibration measurements shown in Figure 1 were performed in quadruplicate (n = 4), and the reported values represent the mean ± standard deviation. The limits of detection were calculated using the standard 3σ/m criterion, where σ represents the standard deviation of blank measurements and m corresponds to the slope of the calibration curve.

The favorable analytical performance of the sensor can be attributed to the combination of selective phosphate extraction by the TBTC-containing membrane, electrochemical preconcentration, background-signal correction, and efficient ion-to-electron transduction provided by the PEDOT: PSS solid-contact layer. These results demonstrate that the developed sensor enables sensitive and quantitative phosphate determination over a broad concentration range, highlighting its potential for tap water-quality monitoring.

### 3.4. Chronoamperometric Response and Potential Monitoring Applications

**Figure 2:**
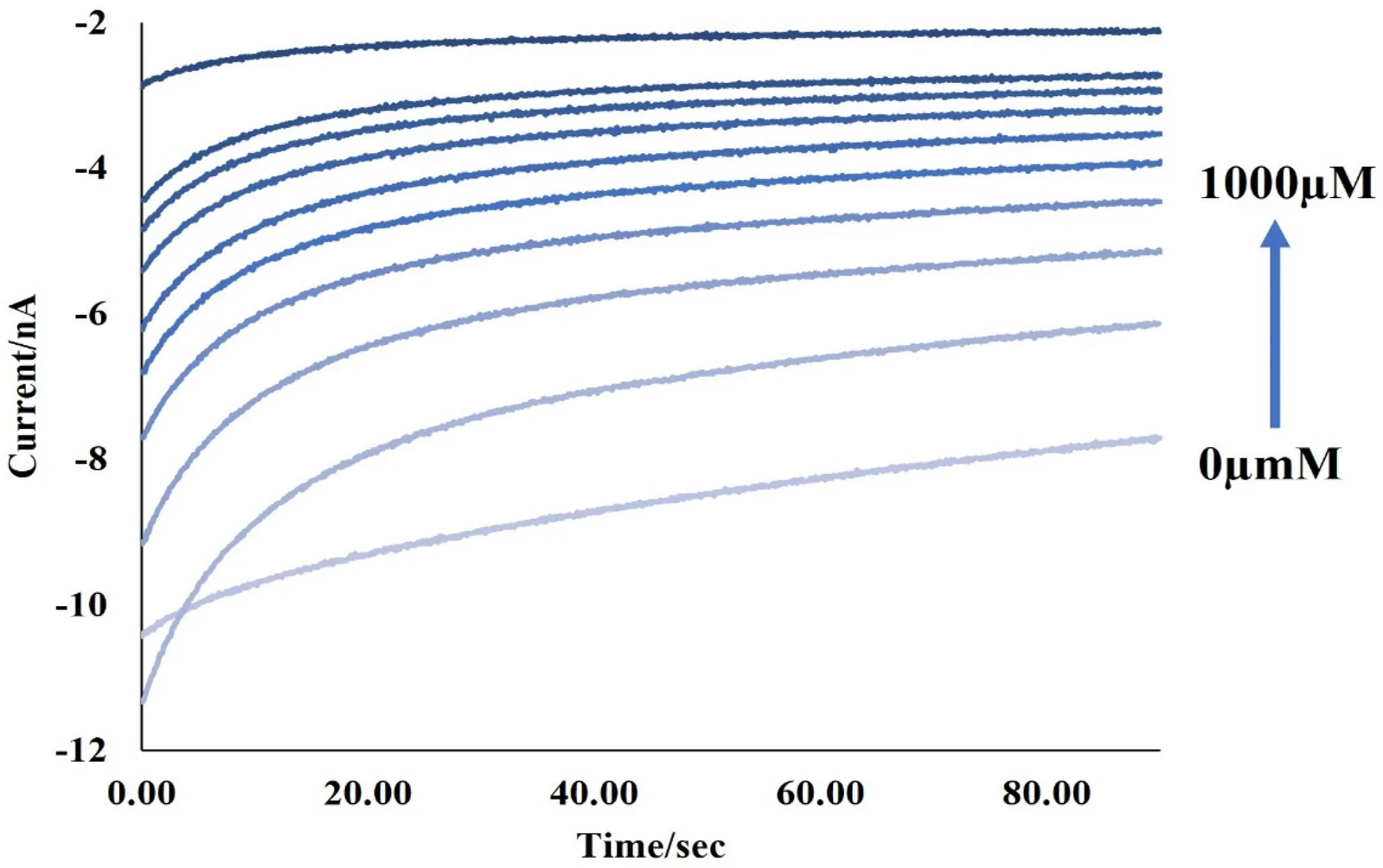
Representative chronoamperometric responses of the PEDOT:PSS/PVC-o-NPOE/TBTC/TDDA phosphate sensor recorded at 0.25 V versus Ag/AgCl for phosphate concentrations ranging from 5 to 1000 μM. The concentration-dependent increase in current demonstrates the capability of the sensor for rapid phosphate determination under chronoamperometric conditions and highlights its potential applicability for future real-time phosphate-monitoring applications.

Chronoamperometric measurements were performed to evaluate the capability of the sensor for rapid phosphate detection under chronoamperometric conditions. Representative current-time responses recorded at an applied potential of 0.25 V versus Ag/AgCl for phosphate concentrations ranging from 5 to 1000 μM are shown in Figure 2.

Following the application of the potential step, an immediate increase in current was observed, followed by a gradual stabilization toward a steady-state value. This behavior is characteristic of ion-transfer processes at membrane interfaces and reflects phosphate transport across the membrane-solution interface and subsequent equilibration within the membrane phase.

As shown in Figure 2, the magnitude of the current response increased systematically with increasing phosphate concentration, producing well-resolved current-time profiles throughout the investigated concentration range. The steady-state current exhibited a clear concentration dependence, demonstrating the ability of the sensor to quantify phosphate directly without the preconcentration and stripping steps required for ITSV measurements.

Notably, measurable current responses were observed even at low phosphate concentrations, indicating the suitability of the sensing platform for detecting environmentally relevant phosphate levels. In addition, the chronoamperometric signals exhibited good reproducibility and stable baselines with minimal drift during the measurement period. The stable response can be attributed to the effective ion-to-electron transduction provided by the PEDOT: PSS solid-contact layer, which promotes efficient charge transfer and minimizes interfacial polarization effects.

Compared with ion-transfer stripping voltammetry, chronoamperometry offers a simpler operational mode that is well suited for rapid measurements and demonstrates the potential applicability of the sensor for future real-time monitoring applications. Although ITSV provides enhanced sensitivity through electrochemical preconcentration, chronoamperometry enables direct phosphate determination with reduced measurement complexity while maintaining a broad working concentration range.

Overall, the chronoamperometric results demonstrate that the PEDOT: PSS/PVC-o-NPOE/TBTC/TDDA sensor provides rapid, stable, and concentration-dependent responses for phosphate determination, supporting its potential applicability for future water-quality monitoring applications.

### 3.5. Analysis of Tap-Water Samples

**Figure 3:**
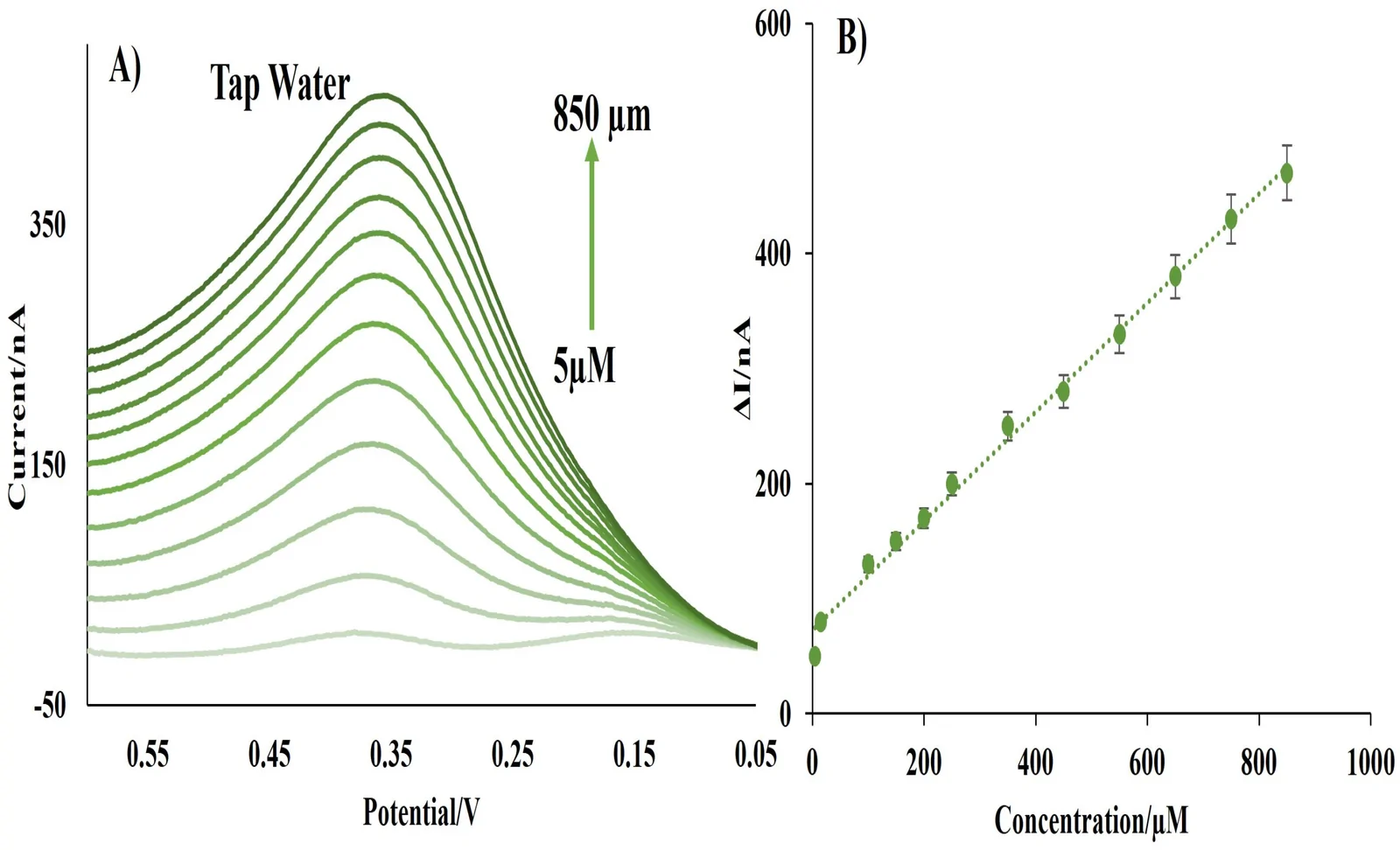
(**A**) Representative ion-transfer stripping voltammograms recorded in tap-water samples containing orthophosphate concentrations ranging from 15 to 850 μM. (**B**) Corresponding calibration curve showing the relationship between background-subtracted stripping peak current (ΔI) and orthophosphate concentration in tap-water samples. Each data point represents the mean value obtained from four replicate measurements (n = 4), and the error bars represent ± one standard deviation (SD). The sensor exhibited high linearity (R^2^ = 0.9966) and a limit of detection (LOD) of 4.7 μM.

The practical applicability of the proposed sensor for phosphate determination was evaluated using tap-water samples to assess its performance in complex real-world matrices. Representative background-subtracted ion-transfer stripping voltammograms obtained for phosphate concentrations ranging from 15 to 850 μM are shown in Figure 3A. Background correction was performed using voltammograms recorded from the tap-water matrix without added phosphate, allowing removal of nonfaradaic currents and matrix-related contributions, and enabling more accurate evaluation of the phosphate-transfer response.

As shown in Figure 3A, well-defined stripping peaks were observed across the investigated concentration range, demonstrating that the phosphate-selective membrane maintained its ion-transfer capability in the presence of naturally occurring ions and dissolved constituents present in tap water. After background subtraction, the stripping current increased proportionally with increasing phosphate concentration, confirming that phosphate ions were selectively extracted and accumulated within the membrane despite the presence of potentially interfering species. Furthermore, the stripping peak potential remained consistent with that observed in the supporting electrolyte, indicating that matrix components did not significantly influence the phosphate-transfer mechanism or the thermodynamics of the ion-transfer process.

Tap-water samples typically contain a complex combination of inorganic ions, organic matter, and other dissolved species that may affect electrochemical measurements. However, the background-corrected voltammograms exhibited highly reproducible peak profiles, stable peak positions, and consistent signal responses throughout the concentration range evaluated. These results confirm that the TBTC-containing membrane provides sufficient selectivity for phosphate recognition in complex matrices. The preservation of a well-defined stripping response after background correction further indicates that the measured analytical signal primarily originates from selective phosphate transfer rather than nonspecific interactions with matrix components.

The calibration curve derived from the background-corrected stripping peak currents is presented in Figure 3B. A strong linear correlation between stripping current and phosphate concentration was obtained over the entire analytical range, with linear regression yielding an R^2^ value of 0.9966. This strong agreement demonstrates reliable quantitative performance of the sensor in tap-water samples. Notably, the calibration behavior observed in tap water was comparable to that obtained in the supporting electrolyte, suggesting that naturally occurring matrix components produced minimal effects on phosphate detection.

The limit of detection (LOD) in tap water was determined to be 4.7 μM, while the limit of quantification (LOQ) was 12.0 μM, representing only a modest increase compared with the 2.8 μM LOD obtained in the optimized supporting electrolyte. The slightly higher detection limit in tap-water samples is likely associated with increased ionic complexity, background fluctuations, and matrix-induced variability at low phosphate concentrations. Nevertheless, the achieved sensitivity remains suitable for monitoring phosphate levels commonly associated with nutrient enrichment in freshwater systems, agricultural runoff, wastewater discharge, and eutrophic environments.

The strong agreement between the background-corrected stripping responses and the corresponding calibration curve confirms that phosphate transfer and membrane accumulation remain quantitative even under complex sample conditions. Importantly, phosphate measurements were achieved without the use of chemical reagents, enzymatic amplification, derivatization steps, or extensive sample preparation. This reagent-free approach provides a simple analytical strategy with potential applicability for future water-quality monitoring applications.

Overall, the tap water analysis demonstrates the robustness and practical applicability of the PEDOT/TBTC-based ion-transfer stripping voltammetry (ITSV) platform. The combination of high linearity (R^2^ = 0.9966), environmentally relevant sensitivity (LOD = 4.7 μM), broad analytical range, and reagent-free operation highlights the potential applicability of this technology for future phosphate-monitoring applications, eutrophication assessment, and early detection of nutrient conditions associated with harmful algal bloom formation.

### 3.6. Selectivity Toward Orthophosphate in the Presence of Competing Anions

**Figure 4:**
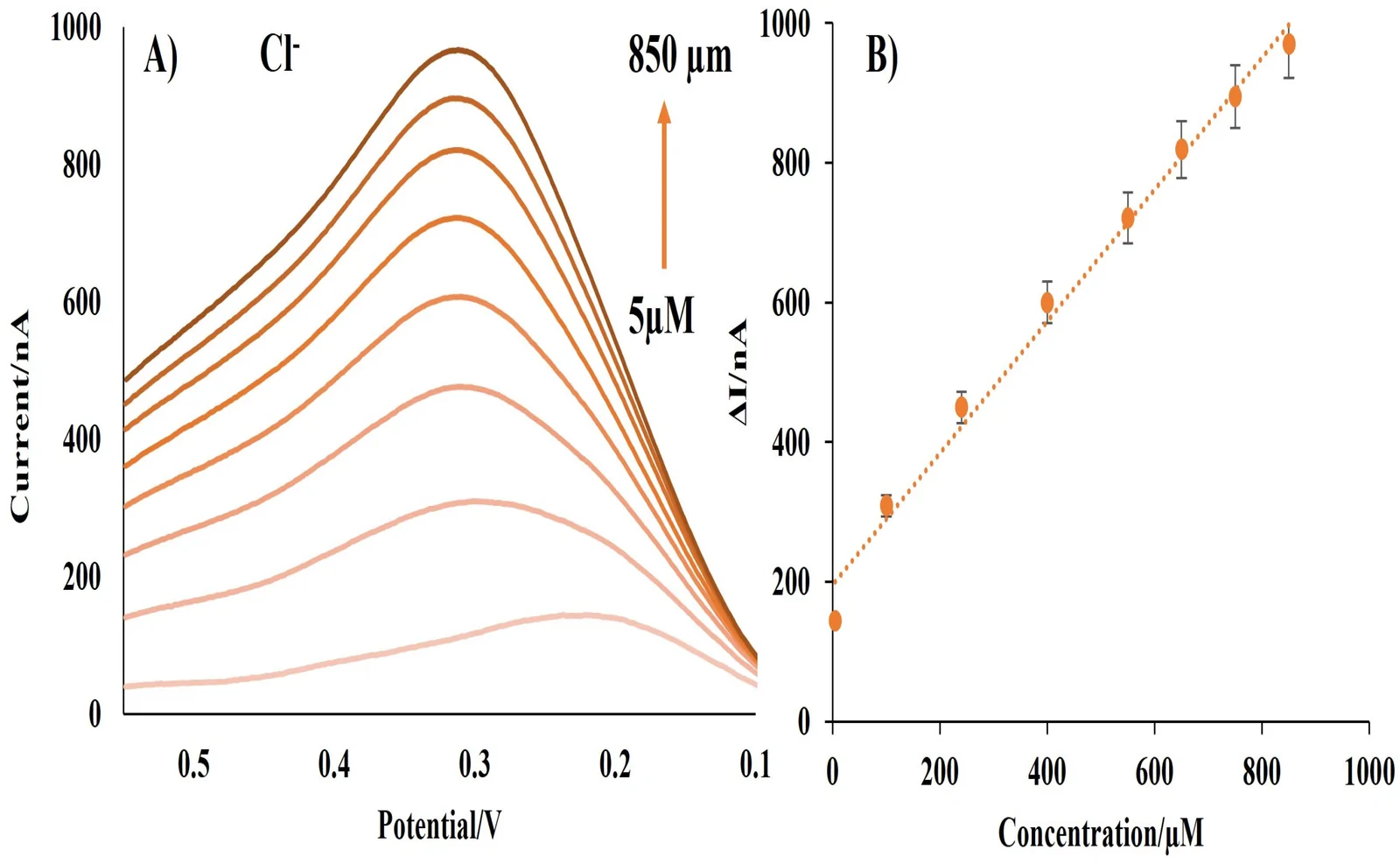
(**A**) Representative ion-transfer stripping voltammograms obtained for orthophosphate concentrations ranging from 15 to 850 μM in the presence of chloride ions (NaCl). (**B**) Corresponding calibration curve showing the relationship between background-subtracted stripping peak current (ΔI) and orthophosphate concentration in chloride-containing solutions. Each data point represents the mean value obtained from three replicate measurements (n = 3), and the error bars represent ± one standard deviation (SD). The sensor exhibited high linearity (R^2^ = 0.997) and a limit of detection (LOD) of 3.8 μM, indicating minimal influence of chloride ions on phosphate determination under the experimental conditions.

The selectivity of the phosphate sensor was evaluated in the presence of chloride ions, one of the most abundant anions in natural and drinking waters. Representative background-subtracted stripping voltammograms and the corresponding calibration curve obtained for phosphate concentrations ranging from 15 to 850 μM in nitrate-containing solutions are shown in Figure 4A, and the corresponding calibration curve is presented in Figure 4B.

As shown in Figure 4A, a well-defined stripping peak was observed at approximately the same potential as that obtained in the supporting electrolyte, indicating that chloride ions had minimal influence on the phosphate-transfer mechanism. The stripping peak current increased systematically with increasing phosphate concentration, demonstrating continued selective accumulation of phosphate within the TBTC-containing membrane.

The calibration curve (Figure 4B) exhibited high linearity over the investigated concentration range of 15 to 850 μM, with a correlation coefficient of R^2^ = 0.997. The calculated limit of detection LOD was 3.8 μM, and the LOQ was 11.2 μM, both only slightly higher than those obtained in the optimized supporting electrolyte. These results indicate that chloride ions do not significantly interfere with phosphate determination and confirm the robustness of the sensor in chloride-rich aqueous matrices.

**Figure 5:**
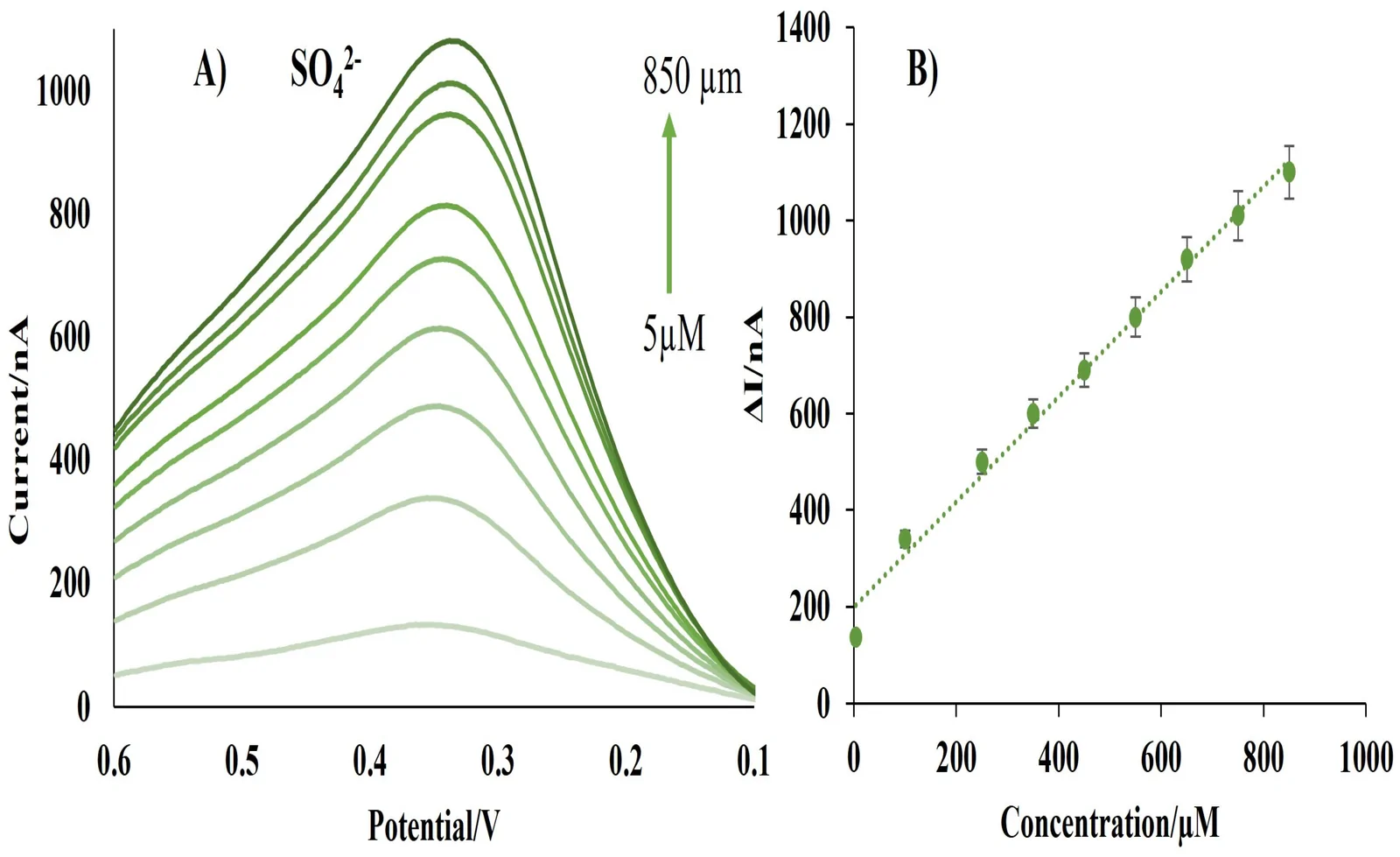
(**A**) Ion-transfer stripping voltammograms obtained for orthophosphate concentrations ranging from 15 to 850 μM in the presence of sulfate ions (Na_2_SO_4_). (**B**) Corresponding calibration curve showing the relationship between background-subtracted stripping peak current (ΔI) and orthophosphate concentration in sulfate-containing solutions. Each data point represents the mean value obtained from three replicate measurements (n = 3), and the error bars represent ± one standard deviation (SD). The sensor exhibited high linearity (R^2^ = 0.9903) and a limit of detection (LOD) of 4.1 μM.

The influence of sulfate ions on phosphate determination was investigated because sulfate is commonly present in tap waters and may occur at concentrations significantly higher than phosphate. Representative voltammograms and the corresponding calibration plot obtained for phosphate concentrations ranging from 15 to 850 μM in sulfate-containing solutions are shown in Figure 5A and Figure 5B, respectively.

Background-subtracted voltammograms exhibited well-defined stripping peaks that increased with increasing phosphate concentration while maintaining a nearly constant peak potential. These observations indicate that sulfate ions do not substantially alter the phosphate-transfer process or hinder phosphate accumulation within the membrane phase.

The calibration curve displayed high linearity over the concentration range of 15 to 850 μM; LOQ was 12.6 μM, with a correlation coefficient of R^2^ = 0.9903; and the corresponding LOD was 4.1 μM. Despite the divalent nature of sulfate, only a minor decrease in analytical performance was observed, demonstrating the strong preferential interaction between phosphate and the TBTC ionophore.

**Figure 6:**
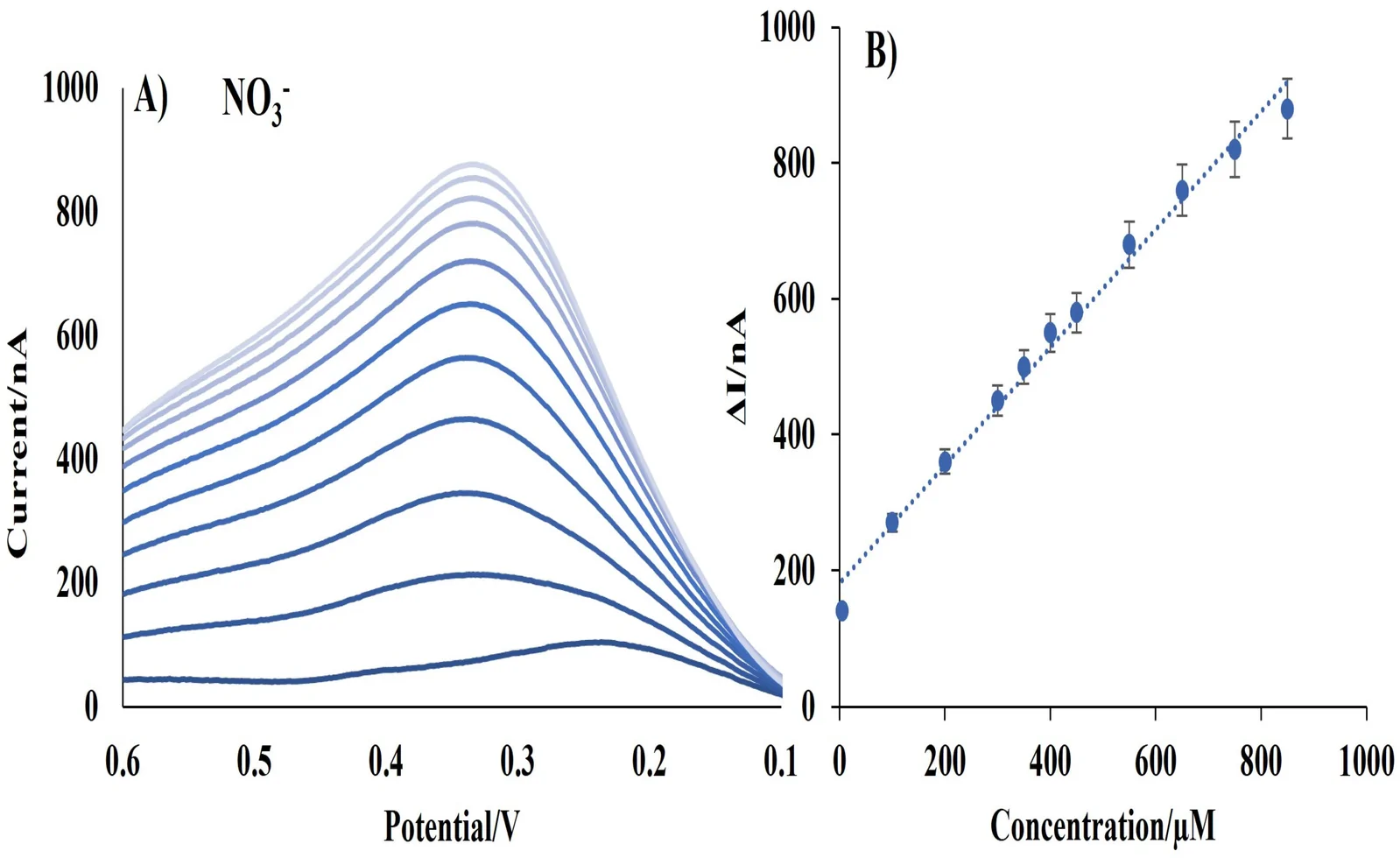
(**A**) Ion-transfer stripping voltammograms obtained for orthophosphate concentrations ranging from 15 to 850 μM in the presence of nitrate ions (NaNO_3_). (**B**) Corresponding calibration curve showing the relationship between background-subtracted stripping peak current (ΔI) and orthophosphate concentration in nitrate-containing solutions. Each data point represents the mean value obtained from three replicate measurements (n = 3), and the error bars represent ± one standard deviation (SD). The sensor exhibited high linearity (R^2^ = 0.992) and a limit of detection (LOD) of 4.7 μM.

The selectivity of the sensor was further evaluated in the presence of nitrate ions, which are frequently encountered in agricultural runoff and wastewater effluents. Representative background-subtracted stripping voltammograms and the corresponding calibration curve obtained for phosphate concentrations ranging from 5 to 850 μM in nitrate-containing solutions are shown in Figure 6A and Figure 6B, respectively.

As observed for chloride and sulfate, the stripping peak current increased progressively with increasing phosphate concentration while the peak potential remained essentially unchanged. The preservation of the voltammetric response indicates that nitrate ions have minimal influence on the phosphate-transfer mechanism and do not significantly compete with phosphate during membrane extraction.

The calibration curve exhibited high linearity over the concentration range of 15 to 850 μM, with a correlation coefficient of R^2^ = 0.992; a detection limit of 4.7 μM was obtained, and the LOQ was 14.1 μM. These results confirm that the sensor maintains reliable phosphate detection in nitrate-containing waters and further demonstrate the selectivity of the TBTC-based membrane toward phosphate.

The interference studies presented herein focused on chloride, sulfate, and nitrate because these ions are among the most abundant inorganic anions encountered in natural waters. While the obtained results demonstrate favorable phosphate selectivity under these conditions, additional investigations involving mixed-ion systems, carbonate/bicarbonate, dissolved organic matter, and humic substances would provide further insight into sensor performance under more complex environmental conditions.

Calibration data shown in Figure 4, Figure 5 and Figure 6 represent the mean ± SD obtained from three replicate measurements (n = 3).

### 3.7. Comparison with Existing Phosphate Sensors

The analytical performance of the proposed ion-transfer stripping voltammetric (ITSV) sensor was compared with representative phosphate sensing technologies reported in the literature (Table 1). The comparison includes conventional colorimetric methods, potentiometric ion-selective electrodes (ISEs), amperometric phosphate sensors, and optical fluorescence-based sensing platforms [8,9,15,16,18,32,33,34].

The molybdenum blue method remains the most widely accepted standard method for phosphate determination because of its excellent sensitivity, high accuracy, and broad applicability to environmental samples [8,9]. However, the method requires multiple chemical reagents, sample preparation procedures, and laboratory-based instrumentation, limiting its suitability for continuous in situ monitoring and autonomous field deployment. Although limits of detection below 1 μM can be achieved, the need for reagent consumption, regular maintenance, and trained personnel increases operational costs and complicates long-term environmental monitoring applications [8,9].

Potentiometric phosphate sensors based on cobalt electrodes and organotin-containing ion-selective membranes provide a reagent-free alternative and offer the advantages of rapid response and straightforward operation [32,33,34]. Cobalt-based phosphate electrodes have been successfully applied to soil extracts over a broad dynamic range, whereas tin-based phosphate ISEs exhibit selective phosphate recognition through organotin ionophores such as tributyltin chloride (TBTC) [33,34]. Nevertheless, these sensors typically rely on open-circuit potential measurements that may suffer from signal drift, temperature sensitivity, and calibration instability during prolonged measurements. In addition, their reported limits of detection are generally around 10 μM [32,33,34], which may restrict their applicability for detecting low phosphate concentrations associated with the onset of eutrophication events.

More recently, solid-state copper/copper phosphate electrodes have demonstrated improved analytical performance with detection limits approaching 1 μM and successful operation in lake water and aquaculture samples [16]. While these sensors represent a significant advancement in all-solid-state phosphate sensing, they remain fundamentally potentiometric devices and therefore do not benefit from electrochemical preconcentration mechanisms that can enhance sensitivity through analyte accumulation prior to measurement [16].

Amperometric molybdate-based phosphate sensors have achieved excellent analytical sensitivity by converting phosphate into electroactive phosphomolybdate complexes that can be quantified using square-wave voltammetry and amperometric techniques [15]. These systems provide low detection limits and successful operation in tap and pond water samples; however, they remain dependent on molybdate chemistry and reagent-assisted detection. Consequently, despite their attractive analytical performance, reagent consumption and chemical derivatization continue to limit their suitability for fully autonomous long-term monitoring systems [15].

Optical fluorescence sensors represent another highly sensitive approach for phosphate analysis. These systems typically utilize luminescent or fluorescence-based recognition probes and can achieve submicromolar detection limits [18]. However, optical sensing platforms generally require excitation sources, fluorescence probes, and dedicated optical instrumentation, which increase system complexity, power consumption, and overall cost. These requirements can present challenges for miniaturization and deployment in remote environmental monitoring applications [18].

In contrast to previously reported phosphate sensing approaches, the sensor developed in this study combines several desirable characteristics that are rarely achieved simultaneously in a single platform. The proposed ITSV sensor operates without chemical reagents, enzymes, redox mediators, optical components, or extensive sample pretreatment. The sensor exhibited a broad linear range of 10–850 μM, a low detection limit of 2.8 μM, high calibration linearity (R^2^ = 0.9954), and successful application to tap-water samples. Furthermore, the analytical performance was maintained in the presence of chloride, sulfate, and nitrate ions, demonstrating the excellent selectivity of the TBTC-containing membrane toward phosphate.

A particularly important distinction of the present work is that the sensing mechanism is based on the direct electrochemical transfer of phosphate ions across a selective membrane interface, rather than phosphate-induced redox reactions, molybdenum-blue chemistry, fluorescence generation, or conventional potentiometric potential changes. The incorporation of a PEDOT: PSS solid-contact transducer further improves charge-transfer efficiency, minimizes potential drift, and enhances long-term signal stability while remaining compatible with miniaturized electrode architectures.

To the best of our knowledge, this study represents the first application of solid-contact ion-transfer stripping voltammetry for direct orthophosphate determination. By combining selective phosphate ion transfer, electrochemical preconcentration, reagent-free operation, and potential applicability for real-time monitoring, the proposed sensing platform introduces a new paradigm for phosphate detection and provides a promising foundation for future portable, wireless, and autonomous water-quality monitoring systems. It should be noted that the primary objective of the present study was to demonstrate proof-of-concept operation of a solid-contact ion-transfer stripping voltammetric platform for phosphate detection. Although the results demonstrate promising analytical performance and applicability to environmental samples, additional investigations involving broader environmental matrices, comparison with established reference methods, and long-term field deployment studies would further support future practical implementation.

## 4. Conclusions

A novel solid-contact ion-transfer stripping voltammetric (ITSV) sensor was developed for the reagent-free determination of orthophosphate in tap-water samples. The sensing platform integrates a phosphate-selective PVC/o-NPOE/TBTC/TDDA membrane with a PEDOT:PSS solid-contact transducer, enabling selective phosphate recognition, electrochemical preconcentration, and efficient ion-to-electron signal conversion. Unlike conventional phosphate detection methods based on redox reactions, enzymatic catalysis, or colorimetric assays, the proposed platform provides direct orthophosphate determination through membrane-based ion-transfer processes without the need for additional reagents or extensive sample preparation.

Under optimized conditions, the sensor exhibited a linear response over the concentration range of 10–850 μM, with a correlation coefficient of R^2^ = 0.9955, a limit of detection (LOD) of 2.8 μM, and a limit of quantification (LOQ) of 9.4 μM. Chronoamperometric measurements further demonstrated rapid concentration-dependent responses over the range of 5–1000 μM, supporting the potential applicability of the platform for future real-time monitoring applications. The sensor also demonstrated favorable analytical performance in tap-water samples and in the presence of chloride, sulfate, and nitrate ions, with correlation coefficients greater than 0.990 and detection limits ranging from 3.8 to 4.7 μM.

To the best of our knowledge, this work represents the first demonstration of a solid-contact ITSV sensor for direct orthophosphate determination. By combining selective phosphate transfer, electrochemical preconcentration, and reagent-free operation, the proposed platform establishes a new strategy for phosphate sensing and provides a promising foundation for future portable and autonomous water-quality monitoring technologies.

The primary objective of this study was to demonstrate the feasibility of a solid-contact ITSV platform for phosphate detection. While the results demonstrate promising analytical performance, additional investigations are needed to further evaluate long-term operational stability, sensor lifetime, batch-to-batch reproducibility, and durability under extended operating conditions. Future studies should also include comparison with established reference methods such as the molybdenum blue assay and EPA Method 365.1, evaluation in broader environmental matrices, detailed surface characterization using SEM and AFM, systematic investigation of phosphate speciation and pH effects, and electrochemical characterization using standard redox probes to assess electroactive surface area and charge-transfer properties. These studies will further support the practical implementation of solid-contact ITSV sensors for environmental phosphate monitoring applications.

## Figures and Tables

**Table 1:** Comparison of the proposed ITSV phosphate sensor with representative phosphate sensing technologies reported in the literature.

Sensor Type	Detection Principle	Linear Range (µM)	LOD (µM)	Reagent-Free	Real-Time Monitoring	Samples	Ref.
Molybdenum Blue Method	Colorimetric	0.1–300	0.05–0.5	No	No	Yes	[8,9]
Cobalt-Based Phosphate Electrode	Potentiometric	10–100,000	10	Yes	Yes	Soil extracts	[32]
Tin-Based Phosphate ISE	Potentiometric	10–1000	10	Yes	Yes	Laboratory samples	[33,34]
Amperometric Molybdate Sensor	SWV/Amperometric	1–500	0.15	No	Limited	Tap and pond water	[15]
Copper/Copper Phosphate Solid-State ISE	Potentiometric	1–1000	1	Yes	Yes	Lake and aquaculture water	[16]
Optical Fluorescence Sensors	Fluorescence	0.1–500	0.1	No	Limited	Yes	[18]
PEDOT: PSS/PVC-o-NPOE/TBTC/TDDA Solid-Contact ITSV Sensor	Ion-Transfer Stripping Voltammetry (ITSV)	10–850	2.8	Yes	Potential applicability	Tap water	This work

## Data Availability

The data presented in this study are available from the corresponding author upon reasonable request.
